# S3: School Zone Safety System Based on Wireless Sensor Network

**DOI:** 10.3390/s90805968

**Published:** 2009-07-28

**Authors:** Seong-eun Yoo, Poh Kit Chong, Daeyoung Kim

**Affiliations:** Korea Advanced Institute of Science and Technology / Daejeon 305-701, Korea; E-Mails: seyoo@kaist.ac.kr (S.Y.); chongpohkit@kaist.ac.kr (P.C.)

**Keywords:** WSN, vehicle detection, school zone

## Abstract

School zones are areas near schools that have lower speed limits and where illegally parked vehicles pose a threat to school children by obstructing them from the view of drivers. However, these laws are regularly flouted. Thus, we propose a novel wireless sensor network application called School zone Safety System (S3) to help regulate the speed limit and to prevent illegal parking in school zones. S3 detects illegally parked vehicles, and warns the driver and records the license plate number. To reduce the traveling speed of vehicles in a school zone, S3 measures the speed of vehicles and displays the speed to the driver via an LED display, and also captures the image of the speeding vehicle with a speed camera. We developed a state machine based vehicle detection algorithm for S3. From extensive experiments in our testbeds and data from a real school zone, it is shown that the system can detect all kinds of vehicles, and has an accuracy of over 95% for speed measurement. We modeled the battery life time of a sensor node and validated the model with a downscaled measurement; we estimate the battery life time to be over 2 years. We have deployed S3 in 15 school zones in 2007, and we have demonstrated the robustness of S3 by operating them for over 1 year.

## 1. Introduction

With the research and development advancements in technology relevant to WSNs (wireless sensor networks) such as MEMS (Micro-Electro-Mechanical Systems), low power communication protocols, and low power embedded software, there has been an increase in trials deploying WSN applications including consumer electronics, PC peripherals, home automation, home security, personal healthcare, toys and games, industrial control and monitoring, asset management [1], and intelligent agriculture [2]. In addition, ITS (Intelligent Transport System) and telematics [3–9] have advantages by incorporating WSNs, since sensors can be paved into the road. In this paper, we propose a novel WSN application called School zone Safety System or S3, and report real world experiences from its development and deployment.

Accidents in a school zone have higher fatality rates since most of them involve children. Children may not be able to judge the velocity of vehicles and just cross the road. To reduce the incidence of fatal pedestrian crashes, practices that can obstruct the view of drivers need to be avoided and vehicle travel speeds must be kept low [10]. There are many analyses which demonstrate the relationship between vehicle travel speeds and incidence of fatal pedestrian crashes. Anderson *et al*. analyzed the relationship, and they report the result quantitatively [11].

To measure vehicle speed and detect parked vehicles, vehicle detection technology is required. For vehicle detection, conventionally a wired loop detector is used. Since it is paved almost across a whole lane and is wired, it takes a lot of time and money to be installed and maintained and it is prone to be cut off [12]. There is abundant research dealing with the problem of vehicle detection and speed measurement [9,12,13]. A representative non-paved vehicle detection system is based on radar technology. Radar can detect a vehicle and measure the speed without any interference from road deformities, but the cost is expensive. In addition, since it is highly sensitive to any objects in its detection zone and prone to detect a vehicle in the adjacent lane [12], radar based vehicle detection cannot measure a vehicle speed accurately. Since it may measure the speed of a vehicle in the adjacent lane, it is only suitable to be used to provide warnings to a driver rather than to regulate a speeding vehicle by capturing it with a speed camera. Pham *et al*. modeled the combined induced magnetic field and the permanent magnetic field of a vehicle in motion as a moving magnetic dipole [3]. Through computer simulation, they demonstrated vehicle detection and identification based on a small magnetic sensor. Knaian introduced a wireless sensor node to detect vehicles in his master thesis [4]. The sensor node included two sensors to measure the speed and a transceiver using the 915 MHz band to report sensing results to a PC. He designed the sensor package from antenna to system software. In addition, he analyzed the battery life-time and cost to build the sensor package. Sing Yiu Cheung *et al*. published papers on traffic measurement and classification with magneto-resistive sensors [5,6]. They proposed a hill-pattern based vehicle detection and classification algorithm and number of experimental results. Ding *et al*. proposed a signal processing algorithm for vehicle detection based on acoustic and magnetic sensors and described their experimental work [7]. We decided to adopt magneto-resistive sensors and WSN to detect vehicles with higher accuracy, because magnetic field technology has been used and proven with inductance loops for many years, a magneto-resistive sensor and a signal processing algorithm can overcome the detection degradation by weather and other environmental variables, and the sensor node can be deployed quickly and it does not cause long traffic jams [12]. Unlike the previous works, we include a control loop based on WSNs to capture speeding vehicles with a speed camera and propose the whole system architecture and application scenarios for a school zone safety system. In addition, with extensive experiments we demonstrate the performance of S3 through detection ratio, speed measurement accuracy, battery life time, and robustness with real deployments.

S3 consists of a TSN (Telematics Sensor Network) system deployed in the school zone and a management system located on top of the TSN. The TSN includes two sub networks, PCN (Parking/Stopping Control sub network) and SCN (Vehicle Speed Control sub network). PCN is based on the well known ZigBee standard, and is designed to warn and record vehicles parking or stopping within a school zone. Since SCN requires delay bounded communication for speed measurements and speed camera control, we design SCN as a two tiered architecture to separate the delay-sensitive part from the remaining delay-insensitive part. For faster packet delivery, we propose a fast and light weight routing algorithm as well as a level based static addressing scheme for the routing algorithm. The baseline-tracking vehicle detection algorithm (BTDA) was developed for vehicle detection and a distributed speed measurement algorithm was implemented. We deployed the sensor nodes in the roads of our campus, and been monitored the system for about six months to examine the detection performance and the reliability of the system before real deployment in school zones. We also measured the speed accuracy of our system on an abandoned old highway and found the accuracy to be over 95%. We also modeled the battery life-time of a sensor node, T-Sensor-v node, and validated the model by a downscaled measurement of 1/153 capacity of the actual deployed battery. By up-scaling the battery capacity, we estimated that the expected life-time of T-Sensor-v node would be over 2 years. Finally, in 2007 we deployed S3 in 15 school zones. We chose one of them and set up a standard detector to measure the performance of S3 for one day. We evaluated the detection performance and speed measurement accuracy of S3 in the real school zone, and confirmed the results from the experiments in our in-campus and old highway testbeds. The robustness of S3 was also proven by being in operation in 15 school zones for over one year from late 2007.

The rest of this paper is organized as follows. Section 2 describes the system architecture and design in details. Section 3 evaluates the system with extensive experiments in two testbeds and a real deployed site. We conclude with a summary of this paper and the future work in Section 4.

## 2. System Architecture and Design

We describe the system architecture and the detailed design of S3 from the WSN viewpoint. We summarize all the abbreviations used in this paper in the Appendix.

### 2.1. Overall System

**Figure 1.**
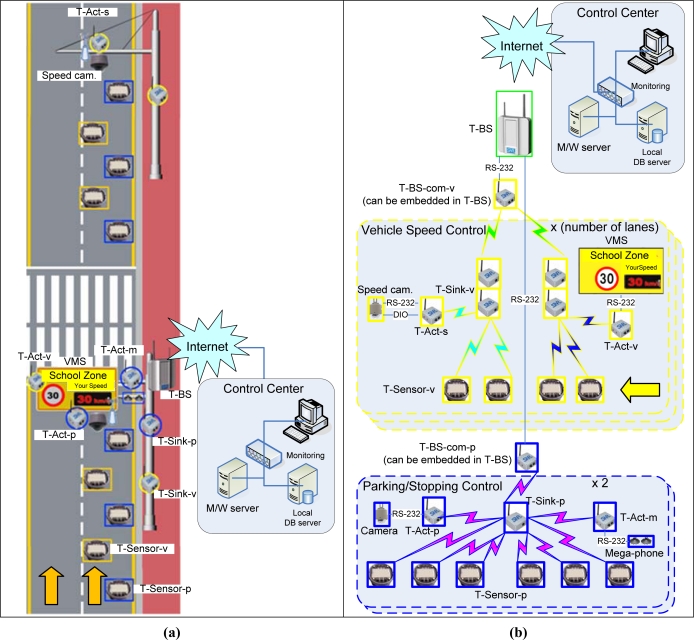
(a) System Description. The direction of a vehicle is from the bottom to the top (yellow arrows). Only one direction is shown in the figure. (b) System Description with network connection. The direction of a vehicle is from the right to the left (yellow arrow). Only one direction is shown in the figure.

S3 consists of a Telematics Sensor Network (TSN) at the bottom and a management system at the top (Figure 1). TSN is divided into two different sub-networks. The first sub-network is to detect vehicles parking or stopping close to a cross-walk [‘Parking/Stopping Control’ in Figure 1(b)]. This sub network controls a loudspeaker module to warn vehicle drivers and a video camera to record the violating vehicles. The other sub-network is to measure the speeds of vehicles passing through the school zone [‘Vehicle Speed Control’ in Figure 1(b)]. Assuming a vehicle is moving from the bottom to the top in Figure 1(a) and from the right to the left in Figure 1(b), the speed is measured at the first set of two T-Sensor-v nodes and displayed in the VMS (Various Messaging System) sign to warn the drivers. The second set of two T-Sensor-v nodes measures the speed again, and the speed camera is used to take photos of speeding vehicles. The management system in the control center consists of a middleware server, a local DB server, and a monitoring terminal. The detailed explanation is presented in the following sub sections.

### 2.2. TSN

TSN is the most basic and important part of S3. It consists of T-BS and two sub network: PCN (Parking/Stopping Control sub network) and SCN (Vehicle Speed Control sub network). In this sub-section, we describe PCN and SCN design details.

#### 2.2.1. PCN

PCN is designed to detect illegally parked or stopped vehicles which hinder other drivers’ visibility of children crossing the road. PCN consists of T-Sensor-p nodes, T-Act-p/m nodes, T-Sink-p nodes, and a T-BS-com-p node, which is attached to the base station, T-BS. ‘p’ stands for ‘parking’ in T-Sensor-p node and T-Sink-p node and T-Act-p node, and ‘m’ is an abbreviation for ‘megaphone’ in T-Act-m node. We adopt the existing ZigBee network for PCN, since PCN does not require any time critical communication. T-Sensor-p node detects a parked or stopped vehicle with an adaptive threshold detection algorithm [14]. Whenever T-Sensor-p node detects an illegally parked or stopped vehicle, it notifies the T-Sink-p node. The T-Sink-p node will then request the T-Act-m node to control the warning system in order to warn the driver. At the same time, T-Sink-p node also sends a command message to a T-Act-p node to begin recording a video of the offending vehicle. If the vehicle does not move within a tolerable time (e.g., 2 minutes) after the warning has been announced, T-Sink-p node reports the event to the Middleware server via T-BS.

#### 2.2.2. SCN

SCN is designed to detect and measure the speed of a passing vehicle. In addition, it controls a VMS (Variable Messaging System) that warns the driver by displaying his speed and a speed camera to capture an image of the violating vehicle.

**Figure 2.**
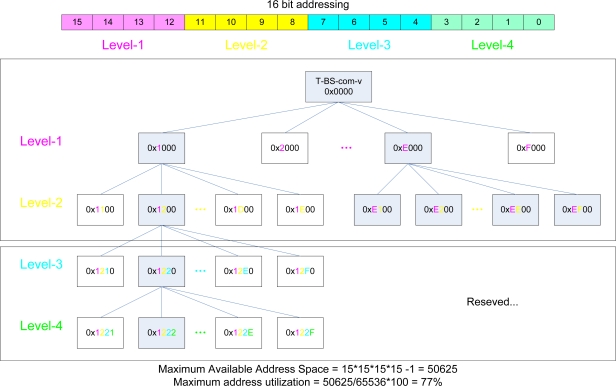
Level based Static Addressing scheme for a fast and light weight routing algorithm for SCN. Each rectagular box including a hexadecimal number represents a sensor node (e.g., T-Sink-v, T-Sensor-v, T-Act-v, etc.) in SCN, and the hexadecimal number is the assigned address for each node.

The communications between T-Sensor-v node, T-Sink-v node, and T-Act-v/s node are time-sensitive and need to be bounded. ‘v’ means ‘velocity’ in T-Sink-v node and T-Sensor-v node or ‘VMS’ for T-Act-v node, and ‘s’ is an abbreviation of ‘speed camera’ in T-Act-s node. However, the communication link between T-Sink-v and T-BS-com-v does not need to be delay-bounded. Therefore, we propose a two-tiered architecture by separating the lower tier of T-Sensor-v node, T-Act-v/s node, and T-Sink-v node from the upper tier of T-Sink-v and T-BS-com-v. If a vehicle passes two consecutive T-Sensor-v nodes, both nodes detect the event and send two DETECT packets to T-Sink-v node. In the VMS region [the lower half of Figure 1(a)], T-Sink-v node sends a SPEED packet to T-Act-v to control the VMS. In the speed camera region, according to the measured speed, T-Sink-v node may send a CAPTURE packet to T-Act-s to direct the speed camera to capture the speeding vehicle. Since a vehicle is detected consecutively by two T-Sensor-v nodes, two DETECT packets from T-Sensor-v nodes can not collide with each other. Since the latency between the DETECT event from the second T-Sensor-v and a command packet (SPEED or CAPTURE) from T-Sink-v is much smaller than the time between two consecutive vehicles, they can not interfere with each other. In addition, we use separate radio channels for VMS and speed camera regions so as not to interfere with each other. For addressing, we used level based static addressing for a fast and robust routing algorithm. 16 bit addresses are used and each nibble is allocated to each level as in Figure 2. We trade off additional addressing space (Figure 2), for simplicity and speed, and a tableless routing algorithm. T-BS-com-v node is in level 0, T-Sink-v node is in level 1 and T-Sensor-v node and T-Act-v node are in level 2. For example, whenever a node with address 0x0000 is asked to send a packet to 0x1200 (at level 2), it can easily know that the next hop address is 0x1000 at level 1, and it forwards the packet to 0x1000. Then 0x1000 sends the packet to its immediate child 0x1200 at level 2.

**Figure 3.**
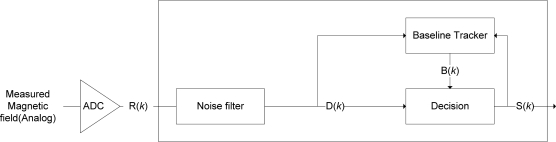
Baseline Tracking Detection Algorithm block diagram.

**Figure 4.**
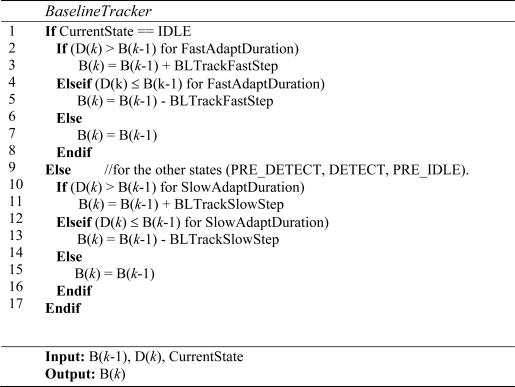
*BaselineTracker* procedure.

**Figure 5.**
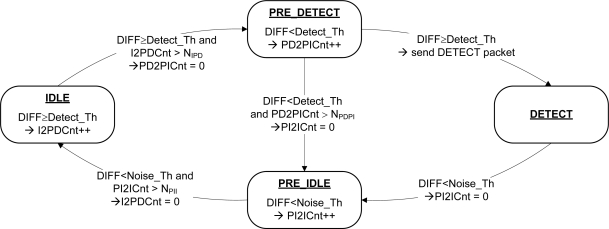
State Diagram for the *decision* block.

Since T-Sensor-v node is battery-operated, a low-power management system and detection algorithm is required. We determined the sampling period (Ps) after considering the maximum detectable speed and the minimum detection length of a vehicle. Most of the time, the main MCU (Microtroller Unit) is in sleep mode but it wakes up every sampling period and runs a baseline-tracking vehicle detection algorithm (BTDA, Figure 3). BTDA consists of a *Noise filter* block, a *Baseline Tracker* block, and a *Decision* block. Although noise is filtered by the hardware before the analog to digital converter (ADC), we adopt a moving average filter as a noise filter to filter out the remaining noise. A sample filtered signal D(*k*) is shown in Figure 8. Since the environmental magnetic field drifts due to environmental factors such as temperature change and sun rise or set, the baseline magnetic level when there is no vehicle over a T-Sensor-v node must be adapted to maintain sensitivity. The *Baseline Tracker* block performs the base line adaptation and Figure 4 shows its pseudo-code. We differentiate the adaptation rates depending on the current state so as not to adapt the baseline to the magnetic field of passing vehicles. When no vehicles are detected, the procedure quickly tracks the baseline. The *Decision* block takes the difference between the magnetic field and the baseline with DIFF = |D(*k*)-B(*k*)|, runs a state machine based decision algorithm (Figure 5), and decide whether or not there is a passing vehicle. There are two thresholds, Detect_Th and Noise_Th to provide hysteresis. Detect_Th is used to decide if a vehicle is detected, while Noise_Th is to release the current state to IDLE (no vehicle) state. When a vehicle is approaching and DIFF is equal to or larger than Detect_Th for the given time (time unit is in Ps) N_IPD_, the current state is changed from IDLE to PRE_DETECT. If DIFF is equal to or larger than Detect_Th in PRE_DETECT, DETECT packet is transmitted to T-Sink-v node and the state is changed to DETECT. When the vehicle passes through and DIFF becomes less than Noise_Th, the state is changed to PRE_IDLE state. If DIFF is less than Noise_Th for the given time N_PII_, the state returns to IDLE. When a vehicle is passing over a magnetic sensor, depending on the type of vehicle, there may be some points when the net magnetic field is the same as the baseline. Counters I2PDCnt, PD2PICnt, and PI2ICnt are used to prevent multiple detections of the same vehicle in this case.

**Figure 6.**
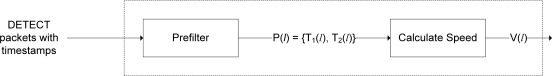
Speed measurement Algorithm block diagram.

T-Sink-v node calculates the speed of vehicles with the help of two T-Sensor-v nodes (Figure 6). Whenever it receives a DETECT packet from each T-Sensor-v node, the *Prefilter* block filters out unbalanced packets which are received in reverse order or incomplete pairs. If *Prefilter* block matches the *l*-th pair of two DETECT packets from two T-Sensor-v node correctly, P(*l*) = {T_1_(*l*), T_2_(*l*)} is passed to the *Calculate Speed* block. T*_i_*(*l*) denotes the DETECT packet reception time from the *i*-th T-Sensor-v node. The *Calculate Speed* block calculates the speed V(*l*) using (distance between two T-Sensor-v nodes)/|T_1_(*l*) − T_2_(*l*)|.

Since sensor nodes in SCN need more computation power and faster wakeup time than those in PCN, we designed different hardware platforms for SCN and PCN. Table 1 summarizes hardware platforms and the development environment.

### 2.3. Management System

All the information such as traveling speed, battery-level, and illegal parking are gathered by the management system through T-BS, which is operated in an industrial PC and attached with T-BS-com-v and T-BS-com-p. The management system consists of middleware server, local DB server, and monitoring terminal. The middleware server gathers the information from each school zone and forwards them to a local DB server. The local DB server saves the information and responds to the request from the monitoring terminals. Since TSN reports low battery warnings, the management system can notify the maintenance team and let them schedule the maintenance proactively.

## 3. Implementation and Evaluation

In this section, we share our experience from developing S3 and performance evaluation focusing on SCN. Our experiments were done in three phases: testbed on our campus at KAIST-ICC (Korea Advanced Institute of Science and Technology - IT Convergence Campus), testbed on an old highway, and a real deployment. With those testbeds, we evaluated detection performance, stability, and the speed accuracy of S3. Finally, we validate our proposed battery life time model with downscaled measurement and estimate the battery lifetime for the real deployed system.

### 3.1. Testbed in ICC: Functional Test and Aging Test in ICC Testbed

**Figure 7.**
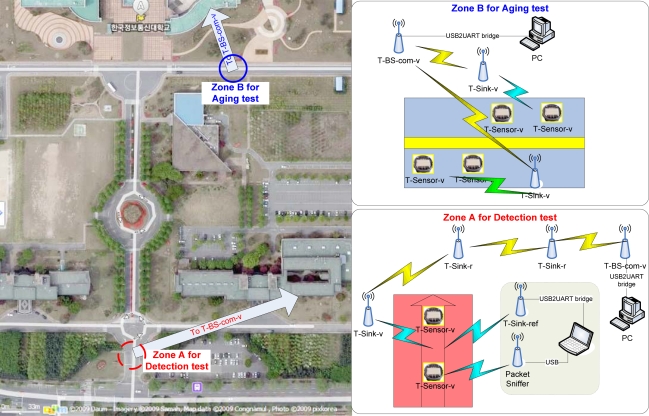
ICC testbed. It consists of two zones. The first zone is located at the main gate of ICC, and the second zone is deployed at the main road in front of the research wing of ICC. The sky view is provided by Daum Communications (http://map.daum.net/).

We prepared an in-campus testbed, ICC testbed, to do basic functional tests on August 2007 (Figure 7). The testbed consisted of two zones. The first zone (‘Zone A’ in Figure 7) is located at the main gate of ICC, and cars passing this zone are moving at less than 30 km/h. We embedded two T-Sensor-v nodes and deployed a T-Sink-v node (Figure 7). The gathered information was delivered to T-BS-com-v through two T-Sink-r nodes (relay nodes) in multi-hop fashion. This zone was intended to evaluate basic functions such as detection and transmission. The second zone (‘Zone B’ in Figure 7) was prepared at the main road in front of the research wing of ICC where cars are moving faster. The road has one-lane in each direction. Two T-Sensor-v nodes were embedded in each lane for each direction, and a T-Sink-v node was hung in a street light on each side (Figure 7). We used this zone to measure stability and the overall performance in the long-term.

**Figure 8.**
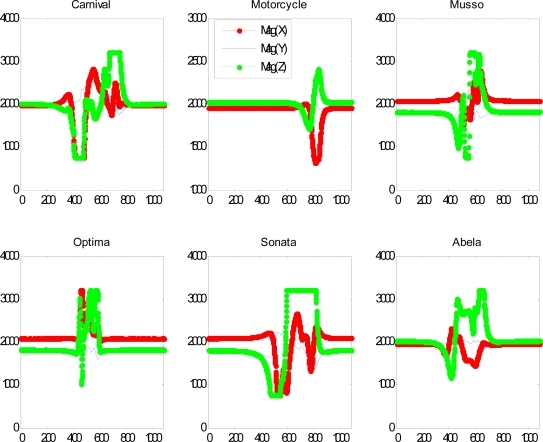
Magnetic Field distortion by different vehicles. Carnival is a kind of van, and Musso is a kind of SUV. The others are categorized as passenger cars.

When a car passes by, the magnetic field measured by a T-Sensor node is distorted, as shown in Figure 8. Depending on the make of each vehicle, the distortion is different. We developed a state-machine based detection algorithm BTDA and evaluated the performance of the detection algorithm in the first zone at the main gate of ICC. For comparison, we set up a reference measurement table temporarily as in dotted-box in Figure 7. A T-Sink-ref node has the same function as a T-Sink-v node, but T-Sink-ref just reports the measured information to the laptop via a serial port rather than via wireless network. T-Sink-ref is used to identify potential problems more accurately. Two T-Sink-r nodes are used to relay any packet between the T-Sink-v node and the T-BS-com-v node. A packet sniffer is used to check the wireless link between T-Sensor-v nodes and the T-Sink-v node. We counted the approaching vehicles and checked whether two T-Sensor-v nodes sent DETECT packets or not with the packet sniffer and a T-Sink-ref node connected to a laptop. From 13:34 to 13:46 on 20^th^ Sep. in 2007, 220 vehicles passed the gate, and among them 218 vehicles passed over two T-Sensor-v nodes, and we recorded the models for 205 vehicles among them.

We summarized the results in Table 2 in two aspects: detection performance (D0-D3) and communication performance (C0-C2). D0 refers to the condition in which two T-Sensor-v nodes detect a vehicle and a T-Sink-v receives the DETECT packets from the two T-Sensor-v nodes, and the T-Sink-v transmits the speed information packet to the first T-Sink-r node. D1 and D2 refer to the conditions in which one or two of T-Sensor-v nodes cannot detect a vehicle, respectively. D3 refers to the situation where two T-Sensor-v nodes detect a vehicle twice. C0 is the condition where the speed measurement of the T-Sensor-v node is delivered to the T-BS-com-v node via two T-Sink-r relay nodes. During multi-hop communication, ACK packets may be lost (the so-called lost ACK problem), and C1 refers to this situation. In this situation, the sender of a data packet retransmits the data packet which has already been received by the receiver, and the receiver receives the packet two times. C2 refers to the situation in which 3 MAC-level retransmissions fail in one of the relay nodes. 98.2% (D0 in Table 2) of vehicles were detected correctly by two T-Sensor-v nodes, and their speeds calculated by the T-Sink-v node were correctly estimated and forwarded to the T-BS-com-v node with a rate of 97.7% (C0 in Table 2).

### 3.2. Testbed on an Old Highway: Speed Measurement Accuracy

**Figure 9.**
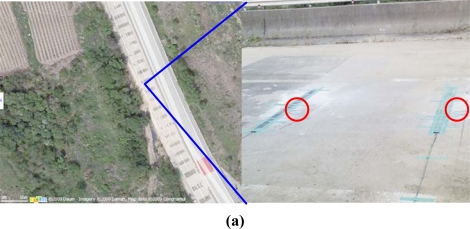

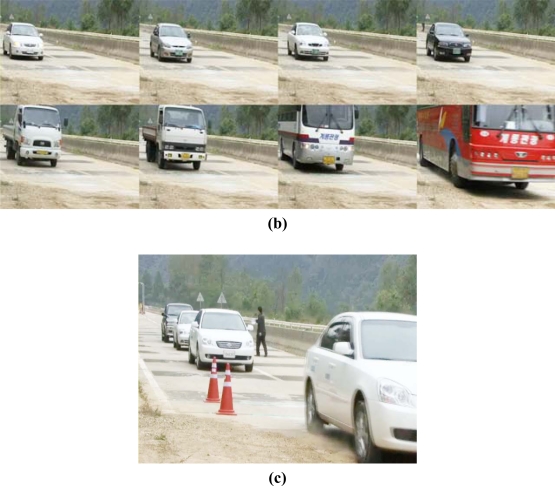
Old highway testbed. (a) Old highway testbed and deployed switching tape and two T-Sensor-v nodes (red circles). (b) Eight vehicles among the nine vehicles used on 12 Oct. in 2007. (c) Four vehicles used on 13 Oct. in 2007.

To evaluate the accuracy of the speed measurement, we set up a testbed on an old highway [Figure 9(a)]. We entrusted the Road Traffic Safety Authority with the measurement tasks. A switching tape based instrument [Figure 9(a)] was used to measure the detection rate and speed. Two T-Sensor nodes were embedded in the pavement, and a T-Sink-v node and a T-BS were deployed. To check the performance of wireless communication, we used an IEEE 802.15.4 packet sniffer from Texas Instruments.

**Figure 10.**
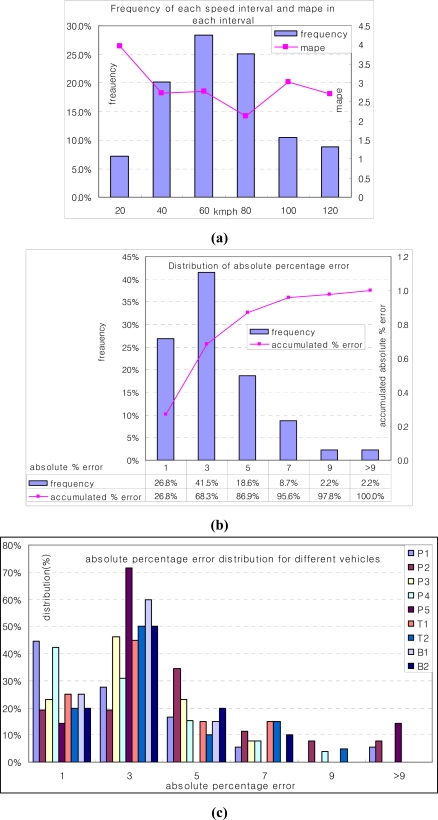
(a) Speed distribution (in the left vertical axis) and MAPE (in the right vertical axis) for each speed interval. (b) Absolute percentage error distribution in measured speed. Horizontal axis represents absolute percentage error. Left vertical axis means frequency of each absolute percentage error. Right vertical axis indicates accumulated distribution of absolute percentage error. (c) Absolute percentage error distribution for different vehicles.

The measurement test was done on October 12–13 in 2007. On 12 October, we measured the speed accuracy for nine vehicles, including two buses, two trucks, and five passenger vehicles, and we showed photos of eight of these vehicles in Figure 9(b). We gathered 183 speed measurements from the detection information of two T-Sensor-v nodes, and they are distributed as the bar graph in Figure 10(a). The accuracy of speed measurement was compared to the standard switching tape based speed measurement instrument in terms of MAPE [Mean Absolute Percentage Error, (1)] for various kinds of vehicles and speeds from 17 km/h to 113 km/h. Two T-Sensor-v nodes detected 183 speeds, but the case when one or two DETECT packets were not delivered to T-Sink-v occurred 10 times due to communication error. Therefore detection performance of T-Sensor-v was 100% (D0 in Table 3), whereas there was 5.5% (10/183 × 100) communication error (C4 in Table 3). In the case of communication errors, we estimated the speeds from the timestamp of each DETECT packet from two T-Sensor-v nodes when the packets were captured by the packet sniffer. Figure 10(a) shows MAPE for each speed interval, and it is generally distributed fairly for different speed intervals. To determine the overall accuracy of the measured speeds, we calculated MAPE for all the measurements and the result was 2.7%:

(1) $$\mathrm{MAPE}=\frac{1}{n}\sum_{i=0}^{n-1}\frac{\left|b_{i}-a_{i}\right|}{a_{i}}\times 100$$

where *a_i_* is taken from the switching tape based speed measurement instrument and *b_i_* is the measured value from SCN.

In addition, we summarized the distribution of absolute percentage error of the measured speed in Figure 10(b) into two aspects: frequency of each absolute percentage error and accumulated distribution of absolute percentage error. The bar graph with the major (left) vertical axis represents the frequency of each absolute percentage error, and the line graph with the minor (right) vertical axis indicates the accumulated absolute percentage error. According to Figure 10(b), 96% of measurements are less than or equal to 7% absolute percentage error. To see the absolute error deviations for different vehicles, we summarize the absolute error for each vehicle in Figure 10(c). The figure shows that the absolute error is concentrated on 3%, but it is independent of different vehicles.

On the next day, 13 October, we evaluated the performance of T-Sensor-v with various scenarios. In the first scenario, we drove four vehicles [Figure 9(c)] on the left edge of the road, and gathered 99 speed measurements with a similar distribution of speeds as the previous test. As in the previous test, the two T-Sensor-v nodes detected the vehicles 100% (Table 3) of the time, but T-Sink node did not calculate the speeds six times, resulting in 6.1% (6/99 × 100) communication error. MAPE was calculated as 3.4% which was slightly more than the MAPE of the test on 12 Oct. The second scenario was to check the performance when vehicles were droved on the right edge, we used 12 vehicles and MAPE was calculated to be 3.2%.

### 3.3. Real Field Deployment

**Figure 11.**
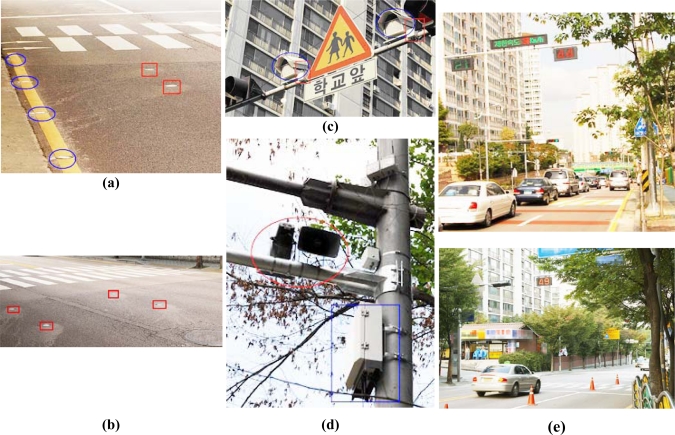
S3 in real school zones. (a) 4 T-Sensor-p nodes and 2 T-Senor-v nodes. (b) 4 T-Sensor-v nodes in two lanes. (c) Speed camera (blue circle) and T-Act-s (red rectangle). (d) T-BS (blue rectangle) and loud speakers (red circle). (e) VMS is notifying that the speed limit is 30 km/h and the current measured speed is 21 km/h (in green), 44 km/h and 49 km/h (in red).

We deployed S3 in 15 school zones in Seoul and Gyeonggi-do during October/November 2007 (Figure 11). We chose one site to evaluate the performance of S3 in a real school zone. As in the experiments described in Section 3.2, we entrusted the Road Traffic Safety Authority with the measurement task.

**Figure 12.**
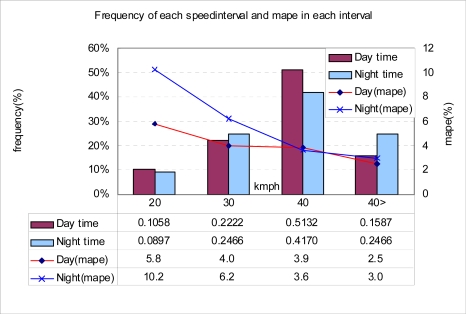
Speed distribution and MAPE for each speed interval.

In the test site, there were two lanes [Figure 11(b)] in each direction where S3 was deployed. For comparison, the standard tape switch based speed measurement instrument was set up. The test was done on 20^th^ Oct. in 2007. For two lanes, the measurement was performed two times, day time (before noon) and night time (from 7 pm). Radio communication of T-Sink-v node for the lane beside the walking road was severely interfered by poles of street lamps and trees by the side of the road, and we could gather only 76% and 70% of packets for day time and night time, respectively. The MAPE of detected speed by T-Sensor-v nodes was 3.7% for both day time and night time. We summarized the test result in Table 4 and Figure 12 focusing on the lane which was nearer to the center of the road.

Four hundred and twelve vehicles (189 and 223 during the day and night, respectively) passed through the lane and 97.8% of them were detected by both T-Sensor-v nodes. From these, 98.5% measurement data were delivered to T-BS. The MAPE of detected speed were 4.3% and 5.2% for day time and night time, respectively. From this test we found a sensing range difference between two T-Sensor-v nodes when a vehicle is not moving in a straight line above two T-Sensor-v nodes. We filtered out those vehicles by checking the raw data of the detection in DETECT packets. By using this filter, we decreased the MAPE to 3.9% and 4.7% for day time and night time, respectively. To evaluate the performance of the deployed S3, we refer to the performance of a commercial wireless vehicle detection system, Sensys Wireless Vehicle Detection Systems (VDS) of Sensys Networks Inc [15]. While SCN of S3 specializes in measuring vehicle speed and *controlling* a speed camera or VMS via WSN, Sensys Wireless VDS is a general purpose VDS that is designed to measure various traffic statistics such as occupancy, traffic volume, and speed. We compared the accuracy of SCN and Sensys Wireless VDS speed measurement. Since Sensys Networks measured the speed accuracy in 15-minute periods [15], we analyzed our results for different 15-minute periods and compared SCN to Sensys Wireless VDS in Table 5. The MAPE of S3 seems to be better than Sensys Wireless VDS, but it is not easy to compare both systems directly. Since both systems may show different results under different traffic conditions, it is necessary to deploy and evaluate SCN and Sensys wireless VDS at the same lane under the same traffic for a more accurate and fair comparison.

### 3.4. Battery Life Time

Since T-Sensor-v nodes are paved into the road, the battery lifetime is very important in deploying S3. We expanded our previous battery lifetime model in [9] to (2) (Table 6) and validated that our model reflected the real world very accurately by comparing the calculated lifetime with the measured life time. We validate our battery lifetime model by using a downscaled measurement and estimated the final battery lifetime for the real deployment by up-scaling the battery capacity.

(2) $$L=\frac{B}{\mathrm{Cc}+\mathrm{Cs}+\mathrm{Cp}+\mathrm{Cr}+\mathrm{Ci}}$$

**Figure 13.**
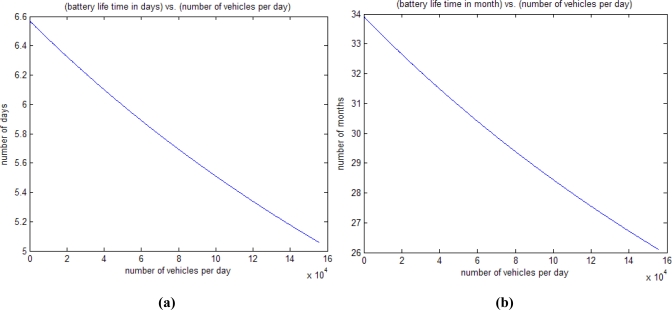
(a) Calculated battery-life time using the measured downscaled battery capacity and the measured current consumption profile of T-Sensor-v node. (b) Estimated battery life time with the actual battery and the measured current consumption profile of T-Sensor-v node.

To calculate the battery lifetime, we measured the actual capacity of the downscaled battery. The actual capacity of the downscaled battery was measured with a constant current consuming load after full charging. The ratio of the measured capacity of the downscaled battery to 80% of the nominal capacity of the actual battery was 1/153. In addition, we measured the power consumption profile of a T-Sensor-v node for different operation modes such as idle, sensing, active (signal processing by MCU), and transmission (MCU + transceiver). Assuming an average traffic per day of up to 155,000 vehicles (1.8 vehicles/second, 154,570 vehicles/day is the average traffic in the busiest highway in Korea from the annual traffic census in 2006), we calculated the battery life time with the model (2) in Figure 13(a). For the maximum traffic 155,000 vehicles/day, the battery life time of T-Sensor-v node was calculated to be 5.06 days [Figure 13(a)].

**Figure 14.**
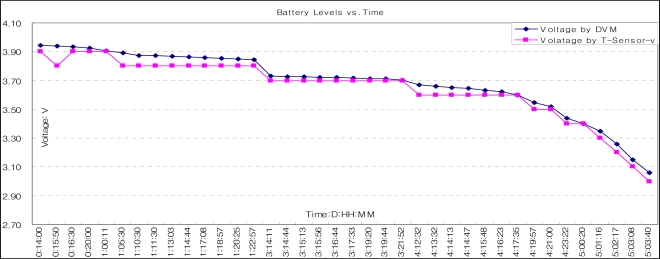
Battery-level (V) vs. time (D:HH:MM). Digital Voltage Meter (DVM) was used to measure the battery-level and T-Sensor-v node itself monitored its battery-level.

To validate the model, we performed measurement with a downscaled battery. We programmed a T-Sensor-v node to detect 1.8 vehicles/second and transmit the detection information through the transceiver, CC2420. We measured the battery level up to 3.0V, which is the minimum level to run a T-Sensor-v node. The result was 5.07 days (5 days 1 hour 40 minutes in Figure 14), while the calculated duration was 5.1 days. We can estimate that the battery life time of T-Sensor-v node will be 5.07 × 153/365 = 2.13 years by up-scaling the battery capacity, and this result is consistent with the battery lifetime from the proposed model [Figure 13(b)].

## 4. Conclusions

To keep children safe in a school zone, reducing vehicle speed and removing obstacles such as illegally parked vehicles that hinders drivers’ views are required. To meet these requirements, we proposed a novel WSN application called School zone Safety System (S3). We developed the baseline-tracking vehicle detection algorithm (BTDA) and implemented a distributed speed measurement algorithm. From the extensive experiments in various testbeds, we evaluated the performance of S3 on the following points: detection performance, speed measurement accuracy, and stability. S3 could detect 100% of vehicles in the testbeds. MAPE of S3 was 3–4% for speeds between 17 km/h and 113 km/h. A battery life time model for T-Sensor-v node was proposed and validated with a downscaled measurement. Both of the model and up-scaling method estimated the battery life time of T-Sensor-v node to over two years which met the design goal of our project. Finally, S3 were deployed in 15 school zones and one of the school zones was used to evaluate the performance of S3 in the real field. Since the first deployment in 2007, S3 has been in operation for over one year in 15 school zones, thus demonstrating their robustness. Topics for future work include the following: enhancing the speed measurement accuracy and radio communication performance, and the development of a solar cell based T-Sink-v node.

## Figures and Tables

**Table 1.** Hardware platforms and development environment for TSN.

		PCN	SCN
MCU		RadioPulse MG2400 (8051Core)	TI MSP430F1611
Transceiver		RadioPulse MG2400	TI CC2420
Magnetic Sensor		AMR Sensor	AMR Sensor

Network Stack		Zigbee stack of RadioPulse	Proprietary Routing/MAC
Compiler		Keil 8051 compiler	msp-gcc compiler

IEEE Sniffer	802.15.4	RadioPulse ZigBee packet analyzer	TI CC2420DK packet sniffer

**Table 2.** ICC gate testbed test results. 98.2% of vehicles were detected by two T-Sensor-v nodes and 97.7% among them is delivered to the T-BS-com-v node.

**Conditions**	**Num. of vehicles**	**Percentage (%)**
D0: 2 T-Sensor-v detecting and T-Sink-v reporting	218−4=214	98.2
D1: 1 T-Sensor-v can’t detect	1	0.5
D2: 2 T-Sensor-v nodes can’t detect	2	0.9
D3: Double detection	1	0.5

C0: Successful end-to-end delivery including C1	214−5=209	97.7
C1: Lost ACK problem in relaying	2	0.9
C2: packet loss in relaying by T-Sink-r	5	2.3
C4: T-Sink-v can’t receive one or two DETECT packets from T-Sensor-v	0	0

**Table 3.** Old highway testbed. 100% of vehicles were detected by two T-Sensor-v node and 94% of them were relayed to the T-BS-com-v node.

**Conditions**	**12 Oct. in 2007**		**13 Oct. in 2007**	
	**Num. of vehicles**	**Percentage (%)**	**Num. of vehicles**	**Percentage (%)**
D0: Two T-Sensor-v detects vehicle	183	100	99	100
C4: T-Sink-v can’t receive one or two DETECT packet from T-Sensor-v	10	5.5	6	6.1

**Table 4.** Real field deployment results. 97.8% of vehicles were detected by two T-Sensor-v nodes and speed for 98.5% of them were calculated and delivered to the T-BS-com-v node.

**Conditions**	**Day time**		**Night time**	

	**Number of vehicles**	**(%)**	**Number of vehicles**	**(%)**
D0: 2 T-Sensor-v detecting	189 − 3 = 186	98.4	223 − 6= 217	97.3
D1: 1 or 2 T-Sensor-v can’t detect	3	1.6	6	2.7

C0: Successful end-to-end delivery	186 − 2 = 184	98.9	217 − 4 = 213	98.2
C4: T-Sink-v can’t receive one or two DETECT packet from T-Sensor-v	2	1.1	4	1.8

**Table 5.** Speed accuracy comparison between SCN and Sensys Wireless VDS [15].

**Sensys [15]**				**S3**							

**11–19 Sep., 2006**				**In the morning on 20 Oct., 2007**				**In the afternoon on 20 Oct., 2007**			

15-Min Quarter Number	Avg. Speed (mph)		Absolute Error (%)	15-Min Quarter Number	Avg. Speed (mph)		Abs. Error (%)	15-Min Quarter Number	Avg. Speed (mph)		Abs. Error (%)
	Laser gun Ground Truth	VDS			Switching tape Ground Truth	SCN			Switching tape Ground Truth	SCN	

2	67.7	67.4	0.4	S1	21.6	21.4	1.1	S1	20.2	20.2	0.0
5	66.7	64.7	3.0	S2	19.5	19.2	1.6	S2	21.6	21.5	0.5
10	64.4	64.4	0.0	S3	18.5	18.3	1.3	S3	20.4	20.2	1
13	66.6	65.9	1.1	S4	20.6	20.4	1.2	S4	20.9	21.2	1.4
17	67.6	65.6	3.0					S5	19.1	19	0.5
21	66.8	66.1	1.0					S6	21.6	21.4	0.9

**MAPE**			**1.4**	**MAPE**			**1.1**	**MAPE**			**0.7**

**Table 6.** Description for each parameter in (2).

**Abbreviation**	**Meaning**
L	Battery life-time of T-Sensor-v node (h)
B	The capacity of battery (mAh)
Cc	Average current consumption for calibrating the sensor block (mA)
Cs	Average current consumption for sensing task (mA)
Cp	Average current consumption for signal processing task (mA)
Cr	Average current consumption for RF transmission task (mA)
Ci	Average current consumption for idle task (mA)

## Appendix

**Table:** A1) Abbreviations defined in the paper.

**Abbreviation**	**Meaning**
BTDA	Baseline-Tracking vehicle Detection Algorithm
PCN	Parking/Stopping Control sub-network
S3	School zone Safety System
SCN	Speed Control sub-network
TSN	Telematics Sensor Network
